# Prevalence and molecular characterization of *Staphylococcus aureus* isolated from goats in Chongqing, China

**DOI:** 10.1186/s12917-017-1272-4

**Published:** 2017-11-25

**Authors:** Zuoyong Zhou, Mengsi Zhang, Hexian Li, Haoyue Yang, Xiaoxia Li, Xinyue Song, Zhiying Wang

**Affiliations:** 1 College of Animal Science, Rongchang Campus of Southwest University, No. 160 Xueyuan Road, Rongchang District, Chongqing, 402460 China; 2 Veterinary Science Engineering Research Center of Chongqing, No. 160 Xueyuan Road, Rongchang District, Chongqing, 402460 China; 3 College of Veterinary Medicine and State Key Laboratory of Agrobiotechnology, China Agricultural University, No. 2 Yuanmingyuan West Road, Haidian District, Beijing, 100193 China

**Keywords:** *Staphylococcus aureus*, Goats, Antimicrobial resistance, Virulence genes, MLST

## Abstract

**Background:**

*Staphylococcus aureus* is an important zoonotic pathogen which not only causes significant economic loss in livestock production but also poses a potential threat to public health. Compared with bovine and swine, the information on the colonization of *S. aureus* in goats is very limited. To understand the prevalence and characteristics of *S. aureus* in goats, we used the nasal swabs collected from apparently healthy goats to isolate *S. aureus*, and tested their antimicrobial susceptibility, virulence gene carrying levels, and multilocus sequence typing (MLST).

**Results:**

In 74 nasal swabs of apparently healthy goats, 32 (43.24%) *S. aureus* strains were isolated and identified, most of which were susceptible to many antibiotics, except for trimethoprim, furazolidone, amoxicillin, lincomycin and roxithromycin, and the resistance incidence of which were 50%, 40.63%, 37.5%, 28.13%, and 21.88% respectively. All the isolates were methicillin-susceptible *S. aureus* (MSSA) and *mecA*-negative. Enterotoxin genes were found in 53.13% of the strains. Of which, *sej* was the most prevalent (21.88%), followed by *seb*, *sec*, and *see* with the same level (18.75%). The most prevalent combination were *seb* + *see* and *seb* + *tst*. None of the *S. aureus* isolates harbored *sea, sed, seh, eta* and *etb*. Multilocus sequence typing (MLST) revealed 6 new alleles (*aroe*-552, *aroe*-553, *glpf*-500, *pta*-440, *yqil*-482 and *yqil*-496) and 5 new sequence types (STs) (3431,3440,3444,3445 and 3461). Using eBURST, the 5 STs were assigned to clonal complex 522 (CC522) and a further CC with no predicted ancestor. Phylogenetic analysis of seven concatenated MLST alleles revealed that the 5 STs were grouped into cluster I composed of *S. aureus* mainly from goats and sheep.

**Conclusion:**

We provide the data for prevalence of *S. aureus* in goats in Chongqing municipality and their characterization which will help in tracking evolution of epidemic strains and their control methods.

## Background

*Staphylococcus aureus* is an important opportunistic pathogen and the cause of infection among human, domestic and wild animals [1–3]. Due to its broad spectrum of inherent virulence factors, the infection of *S. aureus* usually plays an important role for the causing of abscesses, mastitis, pneumonia and meningitis in mammals [4–6]. The invasion of *S. aureus* in domestic animals not only causes significant economic loss in livestock production but also poses a potential threat to public health since these animals can act as the reservoir of methicillin-resistant *S. aureus* (MRSA) [7, 8]. The livestock-associated methicillin-resistant *S. aureus* (LA-MRSA) represented by clonal complex 398 (CC 398) have been shown to be able to colonize and cause serious infections in people having close contact with animals such as veterinarians, farmers and their family members [9–11]. In addition, both handling and consumption of products of these animals colonized by MRSA may provide a potential transmission to humans [12]. Thus, it is of importance to understand the prevalence and characterization of *S. aureus* colonizing the livestock.

In contrast to the studies of *S. aureus* infections in bovine and human, less is known on these bacteria in goats or their relevant products. In 2008, Chongqing municipality was incorporated into the national "Advantage of agricultural products regional planning" in China and recognized as the key areas for beef cattle and goats breeding. The number of live sheep and goats in Chongqing was about 2.26 million and 2.74 million slaughtered was provided by the end of 2015 (Chongqing Statistical Yearbook, 2016). However, there is no investigation on the incidences of colonization in goats by *S. aureus* in Chongqing. To understand the prevalence and characteristics of *S. aureus* in goats, we used the nasal swabs from apparently healthy goats to isolate *S. aureus*, tested their antimicrobial susceptibility and virulence gene carrying levels, and defined multilocus sequence typing (MLST).

## Methods

### Sample collection and *S. aureus* isolation

The nasal swabs were collected from 74 apparently healthy goats from 10 herds in 4 counties (Rongchang, Jiangjing, Zhongxian and Dazu) of Chongqing municipality, transported to the laboratory and stored at 4 °C prior to isolation. The bacteria were enriched in a common broth at 37 °C for 18 h, and then inoculated on 7.5% NaCl agar plates for cultivation at 37 °C for 24 h. The colonies suspected to be *S. aureus* were identified by Gram staining, colony morphology, and coagulase testing. All the presumptive *S. aureus* colonies were then subcultured and confirmed by the PCR amplification of *nuc* (a thermonuclease gene characteristic of *S. aureus*) [13] (Table 1) with the following conditions: denaturation for 4 min at 94 °C, followed by 30 cycles for 1 min at 94 °C, 30 s at 58 °C and 90 s at 72 °C, and a final extension at 72 °C for 3.5 min. *S. sciuri* was used as *nuc*-negative control strain.

**Table 1:** Primers used for *nuc*, *mecA* and virulence genes amplification

Gene Primer	Primer sequence (5′to 3′)	Amplification size (bp)	Reference
*nuc*	SF:GCGATTGATGGTGATACGGTT	279	[13]
	SR:AGCCAAGCCTTGACGAACTAAAGC		
*mecA*	SF: AAAATCGATGGTAAAGGTTGGC	533	[15]
	SR: AGTTCTGCAGTACCGGATTTGC		
*sea*	SF: GGTTATCAATGTGCGGGTGG	102	[16]
	SR: CGGCACTTTTTTCTCTTCGG		
*seb*	SF: GTATGGTGGTGTAACTGAGC	164	[16]
	SR: CCAAATAGTGACGAGTTAGG		
*sec*	SF: AGATGAAGTAGTTGATGTGTATGG	451	[16]
	SR: CACACTTTTAGAATCAACCG		
*sed*	SF:CCAATAATAGGAGAAAATAAAAG	278	[16]
	SR: ATTGGTATTTTTTTTCGTTC		
*see*	SF:AGGTTTTTTCACAGGTCATCC	209	[16]
	SR: CTTTTTTTTCTTCGGTCAATC		
*seg*	SF: TGCTATCGACACACTACAACC	704	[17]
	SR:CCAGATTCAAATGCAGAACC		
*seh*	SF:CGAAAGCAGAAGATTTACACG	495	[17]
	SR: GACCTTTACTTATTTCGCTGTC		
*sei*	SF:GACAACAAAACTGTCGAAACTG	630	[17]
	SR: CCATATTCTTTGCCTTTACCAG		
*sej*	SF: CATCAGAACTGTTGTTCCGCTAG	142	[18]
	SR: CTGAATTTTACCATCAAAGGTAC		
*tst*	SF: ACCCCTGTTCCCTTATCATC	326	[16]
	SR: TTTTCAGTATTTGTAACGCC		
*eta*	SF: ATATCAACGTGAGGGCTCTAGTAC	1155	[20]
	SR: ATGCAGTCAGCTTCTTACTGCTA		
*etb*	SF: CACACATTACGGATAATGCAAG	604	[20]
	SR: TCAACCGAATAGAGTGAACTTATCT		
*pvl*	SF: GTGCCAGACAATGAATTACCC	255	[19]
	SR: TTCATGAGTTTTCCAGTTCACTTC		

### Antimicrobial resistance profile

The *S. aureus* isolates were used for antimicrobial susceptibility test using disk diffusion method according to Clinical and Laboratory Standards Institute (CLSI) guidelines [14]. Following antibiotics with stated concentrations (μg/disc) were used: cefotaxime (30), ceftriaxone (30), streptomycin (10),kanamycin (30), gentamicin (10),tetracycline (30), chloramphenicol (30),lincomycin (2), macrodantin (300),furazolidone (300), vancomycin (30), norfloxacin (10),minocyline (30), amoxicillin (10), cefoxitin (30),clarithromycin (15), levofloxacin (5),roxithromycin (15),trimethoprim (5) and cefepime (30). The presence of *mecA* (staphylococci methicillin resistance gene) [15] (Table 1) was analyzed by PCR method under the same amplification conditions as stated earlier for *nuc*, ATCC 43300 (*mecA* positive) and ATCC 29213 (*mecA* negative) were used as control strains.

### Detection of virulence genes

DNA extraction was done using a commercial DNA extraction kit (Dalian TaKaRa Biotechnology Co.,Ltd.) following the manufacturer’s instructions. Thirteen *S. aureus* virulence genes including enterotoxins (SEs), *sea*, *seb*, *sec*, *sed*, *see*, *seg*, *seh*, *sei*, *sej* [16–18]; cytotoxin *pvl* [19]; exfoliative toxins (ETs), *eta, etb* [20]; and a toxic shock syndrome toxin 1(*tst*) [16] (Table 1) were selected for PCR with the following amplification conditions: initial denaturation for 5 min at 94 °C, followed by 30 cycles of 94 °C for 1 min, 50 °C for 1 min (55 °C for *seg* and *pvl*), and 72 °C for 1 min, with a final elongation of 72 °C for 10 min. *S. aureus* reference strains including ATCC 25923 (*pvl* positive) and ATCC 29213 (*sea*, *sec*, *see* and *tst* positive).

### Multilocus sequence typing (MLST) of *S. aureus*

A random selection of 13 *S. aureus* isolates were analyzed by MLST. The 7 housekeeping genes, carbamate kinase (*arcc*), shikimate dehydrogenase (*aroe*), glycerol kinase (*glpf*), guanylate kinase (*gmk*), phosphate acetyltransferase (*pta*), triosephosphate isomerase (*tpi*), and acetyl coenzyme A acetyltransferase (*yqil*) (http://saureus.mlst.net/misc/info.asp), were amplified by PCR method consisting of initial denaturation at 95 °C for 5 min, followed by a total of 35 cycles of 30 s denaturation at 94 °C, 30 s annealing at 55 °C, and 1 min elongation at 72 °C, with a final elongation step of 10 min at 72 °C. PCR products were sequenced both in forward and reverse directions by Shanghai Invitrogen Biotechnology Co., Ltd. The sequences for each locus were compared to the allele sequences through the MLST website (http://www.mlst.net). Isolates were defined by their alleles at the seven loci (allelic profile), and each allelic profile was submitted to the database to obtain a sequence type (ST) number.

### Clonal complexes (CCs) clustering and phylogenetic analysis of *S. aureus* STs

The clustering of STs was performed using eBURST algorithm (http://eburst.mlst.net/) [21] by comparing the present dataset to that of the MLST database of 4066 STs by the end of May 2017, and ran with the default settings. Clonal complexes (CCs) were composed of STs that shared at least six alleles in common and a predicted ancestral ST and its associated single locus variants (SLVs: the variants differ at one of the seven MLST alleles from the ancestor) and double locus variants (DLVs: the variants differ at two of the seven MLST alleles from the ancestor) [3]. Along with the STs obtained in this research, 23 STs from human (ST7, 12,13, 15, 27, 88, 101 and 240), goats and sheep (ST130, 133, 398, 425, 522, 1595, 1740, 1742, 1743, 1758, 1780, 1781, 2011, 2305, 2328) [2, 9, 22, 23] were collected for phylogenetic analyses. The concatenated sequences of all seven MLST alleles for each ST were aligned using the CLUSTAL X program with default parameters followed by manual inspection. MEGA 4.0 was used to construct neighbour-joining trees [24]. Bootstrapping was performed with 1000 replicates.

## Results

### Isolation and identification of *S. aureus*

**Fig. 1:**
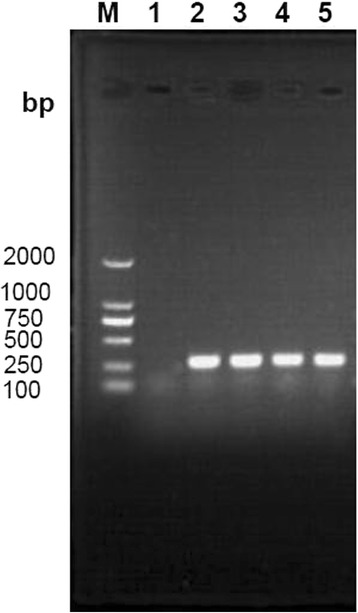
Gel electrophoresis of amplified *nuc* in *S. aureus* isolated from goats in Chongqing. M: DL-2000 DNA marker; 1: *Staphylococcus sciuri*; 2–5: the *nuc* gene of some *S. aureus* isolates by amplified at 297 bp

Of 74 nasal swabs of goats collected from 10 herds, 32 (43.24%) were identified as *S. aureus* through *nuc* amplification (Fig. 1), and all the *S. aureus* isolates were found to be coagulase positive.

### Antimicrobial resistance profile

The antimicrobial susceptibility test showed most of the *S. aureus* isolates susceptible to a majority of antibiotics and the resistance rates below 20%, with the exceptions of following: trimethoprim (50% resistant), furazolidone (40.63%), amoxicillin (37.5%), lincomycin (28.13%), and roxithromycin (21.88%) (Table 2). All the isolates (*n* = 32) were methicillin-susceptible *S. aureus* (MSSA) and cefoxitin-susceptible.

**Table 2:** Results of drug sensitivity test for *S. aureus* (*n* = 32)

Antibiotics	No. of *S. aureus*			Percentage of resistance or sensitive	
	R	I	S	Resistance rate (%)	Sensitive rate (%)
Macrodantin	2	4	26	6.25	81.25
Streptomycin	2	2	28	6.25	87.50
Gentamicin	1	1	30	3.13	93.75
Norfloxacin	4	0	28	12.50	87.50
Kanamycin	2	4	26	6.25	81.25
Vancomycin	3	1	28	9.38	87.50
Ceftriaxone	3	17	12	9.38	37.50
Tetracycline	2	8	22	6.25	68.75
Cefotaxime	1	5	26	3.13	81.25
Chloramphenicol	5	6	21	15.63	65.63
Trimethoprim	16	5	11	50.00	34.38
Cefepime	1	1	30	3.13	93.75
Roxithromycin	7	14	11	21.88	34.38
Levofloxacin	5	0	27	15.63	84.38
Lincomycin	9	10	13	28.13	40.63
Minocyline	0	4	28	0	87.50
Cefoxitin	0	1	31	0	96.88
Clarithromycin	5	2	25	15.63	78.13
Amoxicillin	12	4	16	37.50	50.00
Furazolidone	13	8	11	40.63	34.38

“R” represents resistance; “I” represents intermediate; “S” represents susceptible

### Distribution of virulence genes among *S. aureus* isolates

**Fig. 2:**
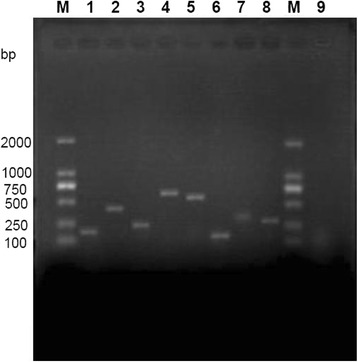
Gel electrophoresis of amplified virulence gene products of *S. aureus* isolates from goats in Chongqing. M: DL-2000 DNA marker; 1: *seb* (164 bp); 2: *sec* (451 bp); 3: *see* (209 bp); 4: *seg* (704 bp); 5: *sei* (630 bp); 6: *sej* (142 bp); 7: *tst* (326 bp); 8: *pvl* (255 bp); 9: amplification of *seb* in *Staphylococcus sciuri*

Since every *S. aureus* carries several virulence genes, but here 19/32 strains carried at least one of the small chosen set of virulence genes (Fig. 2). Enterotoxin genes were detected in majority (53.13%) of the strains, and *sej* gene was found in 7 (21.88%), followed by *seb*, *sec* and *see* in 18.75% isolates each, *sei* (9.38%), *seg* (3.13%). In addition, both *tst* gene and *pvl* gene were detected in 6 (18.75%) isolates. The most prevalent combination was determined to be *seb + see* and *seb + tst* in 12.5% of all isolates (Table 3). There were 8 isolates which only encode a virulence gene.

**Table 3:** Distribution of virulence genes among *S. aureus* isolates from goats (*n* = 32)

Gene	Number of isolates	Detection rate(%)	Genes	Number of isolates	Detection rate(%)
*seb*	6	18.75	*seb + see + pvl*	2	6.25
*sec*	6	18.75	*seb + tst + pvl*	2	6.25
*see*	6	18.75	*seb + see + sej*	2	6.25
*seg*	1	3.13	*seb + see*	4	12.50
*sei*	3	9.38	*see + sej*	3	9.38
*sej*	7	21.88	*seb + tst*	4	12.50
*tst*	6	18.75	*seb + pvl*	3	9.38
*pvl*	6	18.75	*sec + pvl*	2	6.25
			*sec + tst*	2	6.25

### MLST analysis

After MLST analysis, 6 new alleles (*aroe*-552, *aroe*-553, *glpf*-500, *pta*-440,*yqil*-482 and *yqil*-496) and 5 new STs (ST 3431,ST 3440,ST 3444,ST 3445 and ST 3461) were identified and added to the *S. aureus* MLST database (Table 4). The dominant type of *aroE* was *aroE*-552, which was present in 92.31% (12/13) of *S. aureus* isolates, followed by *pta*-440 and *yqiL*-496, which accounted for 69.23% (9/13) and 61.54% (8/13) respectively. The ST3444 was the most common ST type which accounted for 61.54% (8/13) of the *S. aureus* isolates, followed by ST3445 (2 strains), and ST 3431, ST 3440, ST 3461 which just had one strain.

**Table 4:** The alleles and STs of *S. aureus* isolates from goats in Chongqing

Isolates	MLST allelic profile							ST
	*arcc*	*aroe*	*glpf*	*gmk*	*pta*	*tpi*	*yqil*	
1	8	***552***	135	2	***440***	127	***482***	***3431***
2	18	***552***	***500***	2	7	15	5	***3440***
3	8	***552***	135	2	***440***	127	***496***	***3444***
4	8	***552***	135	2	***440***	127	***496***	***3444***
5	8	***552***	135	2	***440***	127	***496***	***3444***
6	18	***552***	45	2	7	15	5	***3445***
7	8	***552***	135	2	***440***	127	***496***	***3444***
8	8	***552***	135	2	***440***	127	***496***	***3444***
9	8	***552***	135	2	***440***	127	***496***	***3444***
10	8	***552***	135	2	***440***	127	***496***	***3444***
11	18	***552***	45	2	7	15	5	***3445***
12	8	***552***	135	2	***440***	127	***496***	***3444***
13	8	***553***	45	2	7	15	5	***3461***

The bold-italic numbers indicate new alleles or STs

### Clonal complexes (CCs) clustering and phylogenetic analysis of *S. aureus* STs

**Fig. 3:**
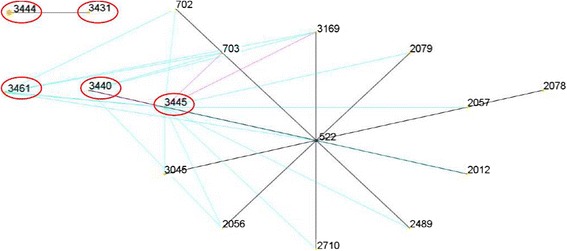
eBURST-generated CC522 containing the major ST522 comprising 14 STs. The primary founder (ST522) is colored blue and positioned centrally in the cluster, and subgroup founders are colored yellow; the SLVs were connected by red lines, and the DVLs were connected by blue lines; the areas of each of the circles indicate the prevalence of the ST in the input data. The novel STs in this study were marked by red oval circle

**Fig. 4:**
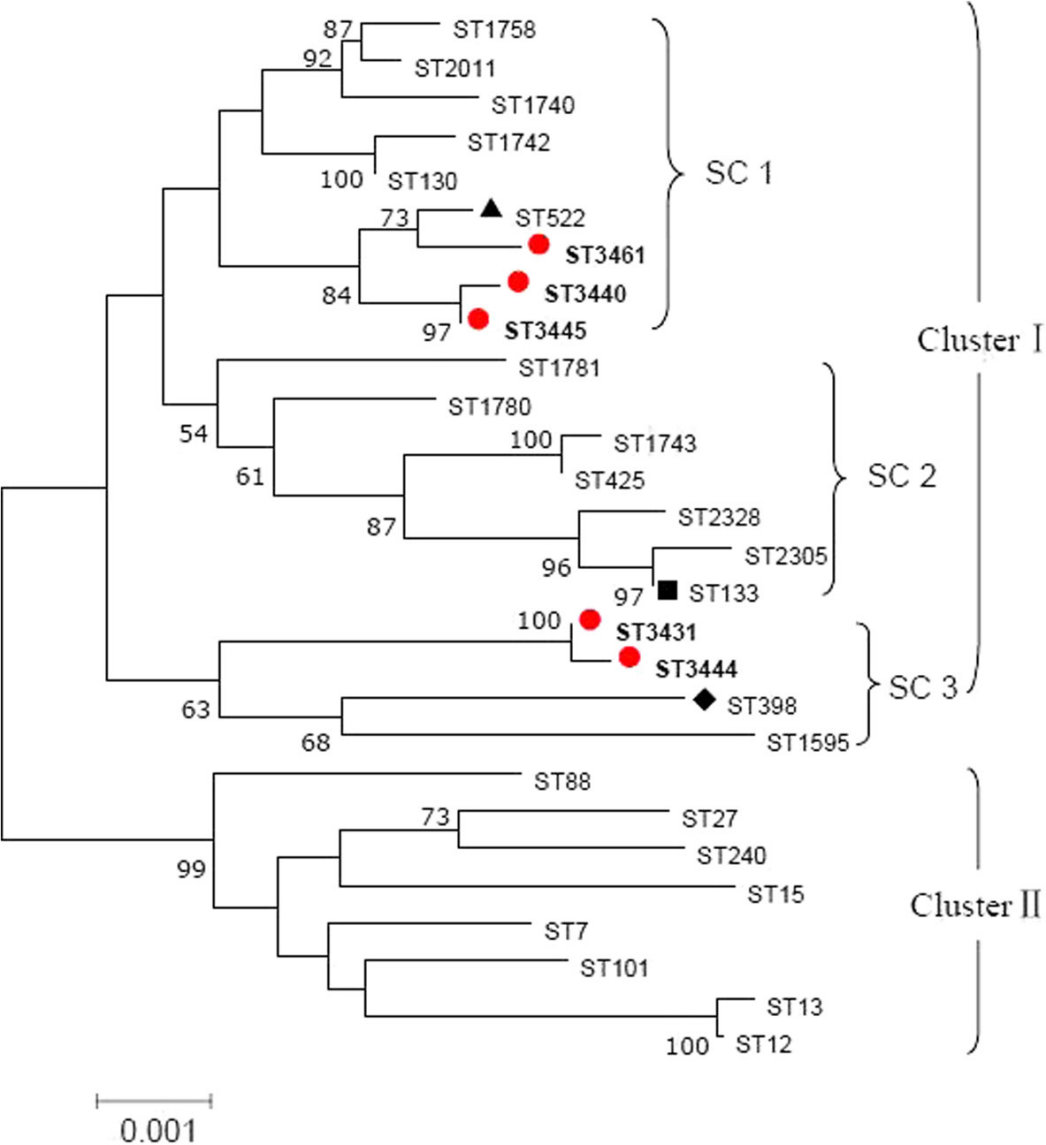
Phylogenetic tree of *S. aureus* STs using the concatenated sequences of the seven MLST genes mainly from human, goats and sheep. ClusterI: mainly from goats and sheep; Cluster II: mainly from human. In clusterI, the STs divided into three sub-clusters (SCs), and the new STs (marked by red filled circles) obtained in this study were clustered in SC1 and SC3. ST398 (marked by black filled diamond), is associated with animals infection [9]; ST522 (marked by black filled triangle) is associated with goats and sheep [2]; ST133 (marked by black filled square) appears to be an ungulate-animal-specific genotype [3]

eBURST generated 103 groups and 483 singletons. The *S. aureus* isolates tested in this study were clustered into CC522 containing ST3440 and ST3445, and a further CC with no predicted ancestor containing ST3431 and ST3444, while ST3461 was a singleton (Fig. 3). The phylogenetic relationship of 28 STs, based on concatenated sequences of seven MLST alleles, resulted in two main clusters: clusterI(mainly from goats and sheep) and cluster II (from humans). In clusterI, there were three sub-clusters (SCs) (SC1 containing ST130, 522, 1740, 1742, 1758, 2011, 3440, 3445 and 3461; SC2 containing ST133, 425, 1743, 1780, 1781, 2305 and 2328; SC3 containing ST 398, 1595, 3431 and 3444) (Fig. 4).

## Discussion

This is the first study of the prevalence of *S. aureus* from the nose swabs of apparently healthy goats in Chongqing. Our results were comparable to the carriage frequency of *S. aureus* in healthy sheep in Tunisia [25] approaching to ~ 45% level, which were higher than reported by other investigators in dairy sheep and goats [26–28], but lower than that in goats in Norway and Denmark [22, 29].

It is interesting that all the *S. aureus* isolates in this study were methicillin-susceptible *S. aureus* (MSSA) since they were sensitive or medium sensitivity to cefoxitin and *mecA* tested negative. The majority of *S. aureus* showed relatively low resistance to vancomycin, ceftriaxone, macrodantin, streptomycin, kanamycin, tetracycline, gentamicin, cefotaxime and cefepime, which is similar to the resistance of *S. aureus* isolated from sheep and goat in Spain [23], but lower than the resistance of *S. aureus* from goats in Taiwan [26]. The relatively high sensitivity spectrum of *S. aureus* from Chongqing is likely due to a limited use of antimicrobials for goats [28].

The infections caused by *S. aureus* is associated with its virulence factors which allow it to adhere to surface, invade or avoid the immune system, and cause harmful toxic effects to the host [30, 31]. More than half of isolates in this study were tested positive for SE genes, which is similar to other reports in *S. aureus* from dairy goats [29] and their milk products [32], and higher than the result in goats and cows affected with mastitis [33]. Of the SE genes tested, *sej* was the most predominantly occurring gene which is different from the detection rates in goats from Taiwan or sheep in Tunisia where there were no *sej* occurrence [25, 26]. Except for *sej*, the *seb*, *sec*, *see*, *tst* and *pvl* carriage rate of 18.75% is similar to the other reports in *S. aureus* from goats and their milk products [26, 32, 34, 35] but lower than *sec* and *tst* found in *S. aureus* from dairy goats [29], or in MSSA from sheep [25]. In contrast to the other report [29], although *sec* and *tst* showed the same detection rates, they were not always co-detected. In the previous study, *see* was identified only in *S. aureus* associated with mastitis in goat [26], the carry level of *seb* and *see* in this study are inconsistent with other reports [32, 35] where there were no *S. aureus* strain harboured *seb* and *see*. *Pvl* is associated with *S. aureus* causing goat subclinical mastitis [36]. Different from the previous report [25], we found 18.75% isolates *pvl-*positive.

To understand the molecular characteristics of *S. aureus* isolates from Chongqing goats, we used MLST typing and found 6 new alleles (*aroe*-552, *aroe*-553, *glpf*-500, *pta*-440,*yqil*-482 and *yqil*-496) and 5 new STs (3431,3440,3444,3445 and 3461). The dominant alleles belonged to *aroE* was *aroE*-552, which occurred in most of *S. aureus* strains, followed by *pta*-440 and *yqiL*-496. In the newly found STs, ST3444 was the most common, which occurred in approximately 60% of the *S. aureus* isolates. It is relatively normal for *S. aureus* from goats to possess novel alleles or STs in contrast to human or bovine strains, which may be simply due to the investigation of a new population, since most reports have focused on human clinical isolates or bovine mastitis [37].

The eBURST analyses assigned *S. aureus* isolates to CC522 containing ST3440 and ST3445, and a further CC with no predicted ancestor. The results from phylogenetic analyses showed that STs from goats and sheep were separated from that of human, thus formed two main clusters. Three new STs (ST3440, 3445 and 3461) were sub-grouped in the branch of SC1 containing ST522, the primary founder ST of CC522, which was reported previously from cases of goat mastitis or goat’s milk samples [2, 3, 23]. ST3444, the predominate ST in this study, and ST3431 were sub-clustered into the branch SC3 containing ST398, which is associated with animals infection [9]. In SC2, ST133 was found in variety of animals including cows, goats and sheep [2, 3, 22, 23], and was found to be the most common animal-associated MLST type [3]. Our results confirmed the relative close relationship of ST 3440, ST3445 and ST3461 with ST522, and ST3444, ST3431 with ST398.

## Conclusion

This is the first study to report the prevalence rate, antimicrobial resistance profile, virulence genes association and MLST characteristics of *S. aureus* from goats in Chongqing municipality. This study will help in tracking evolution of *S. aureus* epidemic strains and proving the methods to control *S. aureus* in goats in China.

## Abbreviations

CC: Clonal complex
DLVs: Double locus variants
ETs: Exfoliative toxins
LA-MRSA: Livestock-associated methicillin-resistant *S. aureus*
MLST: Multilocus sequence typing
MSSA: Methicillin-susceptible *S. aureus*
PCR: Polymerase chain reaction
SEs: Enterotoxins
SLVs: Single locus variants
STs: Sequence types
